# Genetic diversity of *Blastocystis* in kindergarten children in southern Xinjiang, China

**DOI:** 10.1186/s13071-020-3890-0

**Published:** 2020-01-10

**Authors:** Meng Qi, Zilin Wei, Ying Zhang, Qiyuan Zhang, Juanfeng Li, Longxian Zhang, Rongjun Wang

**Affiliations:** 1 College of Animal Science and Veterinary Medicine, Henan Agricultural University, Zhengzhou, 450002 Henan People’s Republic of China; 2 College of Animal Science, Tarim University, Alar, 843300 Xinjiang People’s Republic of China

**Keywords:** *Blastocystis*, SSU rRNA, genetic polymorphism, children, Xinjiang

## Abstract

**Background:**

*Blastocystis* is one of the most common intestinal parasites in humans and various animals worldwide. Few studies are available regarding the genetic characterization of *Blastocystis* infections in humans in China.

**Methods:**

In the present study, 609 fecal samples were collected from two- to six-year-old kindergarten children in southern Xinjiang and were examined by polymerase chain reaction (PCR).

**Results:**

The infection rate of *Blastocystis* was 14.3% (87/609); no significant difference was observed among counties and between sexes. *Blastocystis* subtypes ST1 (*n* = 38), ST2 (*n* = 8), and ST3 (*n* = 41) were identified by sequence analysis of the small subunit ribosomal RNA gene. Genetic polymorphisms were observed at the intra-subtype level, including seven variations for ST1 (ST1A to ST1G), four for ST2 (ST2A to ST2D), and two for ST3 (ST3A and ST3B); with ST1F and ST2B being new variations.

**Conclusions:**

ST1 and ST3 are the two common *Blastocystis* subtypes in the study area. More extensive studies in both humans and animals in different regions are needed to better characterize the transmission of *Blastocystis*.

## Background

*Blastocystis* transmitted by the fecal-oral route, is a strictly unicellular protozoan that inhabits the gastrointestinal tract in humans and animals [1]. Although the pathogenicity of *Blastocystis* remains controversial, its infection is associated with various gastrointestinal disorders, irritable bowel syndrome, and cutaneous lesions [2, 3]. Moreover, patients infected with *Blastocystis* have been observed to be relieved of clinical signs after successful treatment [4–6]. It is estimated that over one billion individuals have been infected with *Blastocystis* throughout the world [7]. Nevertheless, the infection rates of *Blastocystis* vary widely among regions, possibly reaching 30% and 30–76% in industrialized and developing nations, respectively [8–11].

There is extensive genetic variation within the genus *Blastocystis*. Based on molecular analyses of the small-subunit ribosomal RNA (*SSU* rRNA), 17 subtypes (ST1 to ST17) have been described, ST1 to ST9 are identified in both humans and animals, while the others are exclusively identified in animals [12, 13]. ST3 is the most commonly detected subtype in humans, followed by ST1, ST2 and ST4, whereas ST5 to ST9 are rarely found [14, 15]. In addition, genetic diversity varies dramatically among *Blastocystis* subtypes as well as at the intra-subtype level [16–18].

In China, the first report of *Blastocystis* infection in humans was published in 1990 [19]. Since then, a high prevalence and abundant genetic diversity of *Blastocystis* have been found in humans, non-human primates, and domestic and wild animals [20, 21]. So far, six different subtypes (ST1 to ST6) have been identified in humans from 12 provinces, with ST3 being the most dominant subtype [20]. In contrast, *Blastocystis* infection has been identified in animals in eight provinces: 10 known (ST1 to ST7, ST10, ST13 and ST14), and four novel (Novel 1, Novel 2, Novel 3 and Novel 4) subtypes have been reported [20]. Nevertheless, no report is available regarding *Blastocystis* infection in both humans and other animals in Xinjiang Uygur Autonomous Region (hereafter referred to as Xinjiang), China. The aim of the present study was to estimate the prevalence and genetic diversity of *Blastocystis* in kindergarten children in Xinjiang, China.

## Methods

### Sample collection

**Fig. 1:**
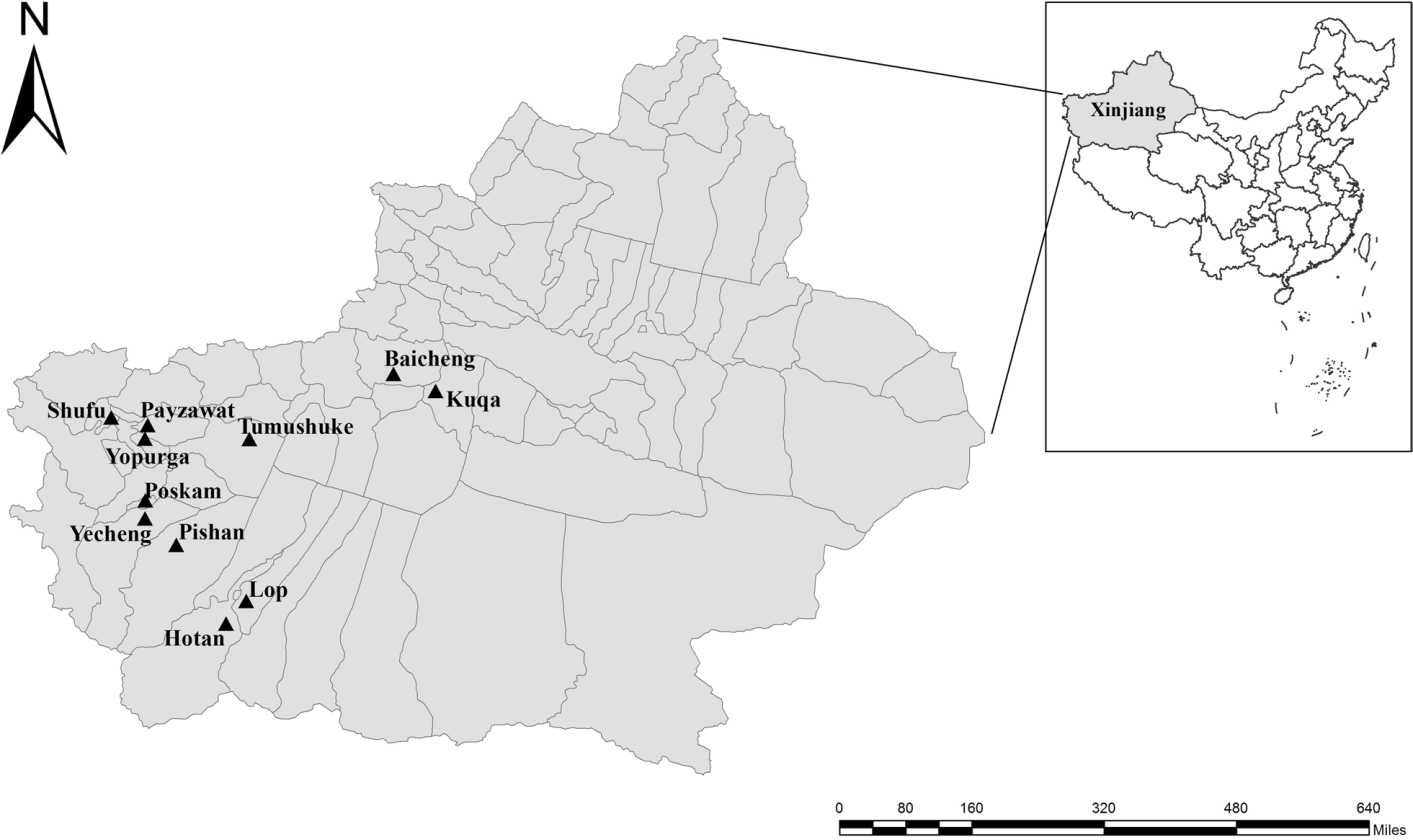
Geographical location of the study. Triangles indicate the sampling locations in southern Xinjiang, China

From August 2017 to January 2019, 609 fresh fecal samples were collected from kindergarten children (two to six years of age) located in Tumushuke, Payzawat, Shufu, Yopurga, Yecheng, Hotan, Baicheng, Poskam, Kuqa, Pishan, and Lop of southern Xinjiang, China (Fig. 1). After being informed of the study purpose and procedures in writing by kindergarten staff, parents or guardians who agreed to their children’s participation were given a plastic fecal collector labeled with a unique number. Fresh stool samples were collected in the morning. No diarrhea was observed during sampling. In total, 609 fecal samples were collected, transported to the laboratory and stored at 4 °C prior to further analysis.

### DNA extraction and PCR amplification

Genomic DNA was extracted from the fecal samples using the E.Z.N.A® Stool DNA Kit (Omega Bio-tek, Inc., Norcross, GA, USA), according to manufacturer-recommended procedures. *Blastocystis* subtypes were determined by PCR amplification of an approximately 600 bp fragment of the *SSU* rRNA gene using the primers RD5 (5′-ATC TGG TTG ATC CTG CCA GT-3′) and BhRDr (5′-GAG CTT TTTA ACT GCA ACA ACG-3′); the PCR reaction conditions were adopted from a previous report [22]. Positive controls (a positive sample already successfully sequenced) were included in each amplification reaction. The PCR products were visualized by electrophoresis in 1% agarose gels (w/v) with GelRed™ (Biotium Inc., Hayward, CA, USA) staining.

### Sequencing and phylogenetic analyses

Positive PCR products were sequenced by Genewiz (Suzhou, China); accuracy was confirmed by two-directional sequencing. Nucleotide sequences were aligned with reference sequences from the GenBank database using the program ClustalX 2.01 (http://www.clustal.org/) to determine the subtypes. Phylogenetic analysis adopted a neighbor-joining model of the aligned sequences implemented in MEGA7 (http://www.megasoftware.net/), with 1000 replicates to assess the robustness of clusters.

### Statistical analysis

The Chi-square test was used to compare *Blastocystis* prevalences. Differences were considered significant when *P* < 0.05.

## Results

### Prevalence of *Blastocystis*

The PCR analysis of 609 fecal samples showed the presence of *Blastocystis* oocysts in 87 samples (14.3%) from 11 counties investigated (Table 1). The highest (23.8%, 15/63) and lowest (5.7%, 2/35) prevalences were observed in Yopurga and Poskam, respectively (*χ*^2^ = 5.14, *df* = 1, *P* < 0.05) (Table 1). There was no significant difference in the prevalence of *Blastocystis* between males (14.7%) and females (13.9%) (*χ*^2^ = 0.09, *df* = 1, *P* > 0.05) (Table 1).

**Table 1:** The infection rate and subtype distribution of *Blastocystis* in kindergarten children at different locations in Xinjiang, China

Collection site	Sex	No. positive/total no. examined (%)	Subtype
Tumushuke	Male	4/33 (12.1)	ST1C (*n* = 1); ST3A (*n* = 2); ST3B (*n* = 1)
	Female	1/29 (3.5)	ST3A (*n* = 1)
	Subtotal	5/62 (8.1)	ST1C (*n* = 1); ST3A (*n* = 3); ST3B (*n* = 1)
Payzawat	Male	0/14 (0)	
	Female	1/11 (9.1)	ST3A (*n* = 1)
	Subtotal	1/25 (4.0)	ST3A (*n* = 1)
Shufu	Male	3/26 (11.5)	ST1B (*n* = 1); ST3A (*n* = 1); ST3B (*n* = 1)
	Female	6/22 (27.3)	ST1D (*n* = 1); ST3A (*n* = 3); ST3B (*n* = 2)
	Subtotal	9/48 (18.8)	ST1B (*n* = 1); ST1D (*n* = 1); ST3A (*n* = 4); ST3B (*n* = 3)
Yopurga	Male	6/34 (17.7)	ST1A (*n* = 1); ST1B (*n* = 1); ST2A (*n* = 1); ST3A (*n* = 3)
	Female	9/29 (31.0)	ST1B (*n* = 1); ST1E (*n* = 1); ST2A (*n* = 2); ST2B (*n* = 1); ST3A (*n* = 3); ST3B (*n* = 1)
	Subtotal	15/63 (23.8)	ST1A (*n* = 1); ST1B (*n* = 2); ST1E (*n* = 1); ST2A (*n* = 3); ST2B (*n* = 1); ST3A (*n* = 6); ST3B (*n* = 1)
Yecheng	Male	7/46 (15.2)	ST1A (*n* = 5); ST3A (*n* = 2)
	Female	9/43 (20.9)	ST1A (*n* = 6); ST3A (*n* = 3)
	Subtotal	16/89 (18.0)	ST1A (*n* = 11); ST3A (*n* = 5)
Hotan	Male	8/40 (20.0)	ST1A (*n* = 3); ST1B (*n* = 2); ST1C (*n* = 1); ST3B (*n* = 2)
	Female	3/40 (7.5)	ST1B (1); ST3A (1); ST3B (1)
	Subtotal	11/80 (13.8)	ST1A (*n* = 3); ST1B (*n* = 3); ST1C (*n* = 1); ST3A (*n* = 1); ST3B (*n* = 3)
Baicheng	Male	2/11 (18.2)	ST3A (*n* = 1); ST3B (*n* = 1)
	Female	1/12 (8.3)	ST3A (*n* = 1)
	Subtotal	3/23 (13.0)	ST3A (*n* = 2); ST3B (*n* = 1)
Poskam	Male	1/17 (5.9)	ST1C (*n* = 1)
	Female	1/18 (5.6)	ST1A (*n* = 1)
	Subtotal	2/35 (5.7)	ST1A (*n* = 1); ST1C (*n* = 1)
Kuqa	Male	3/19 (15.8)	ST3A (*n* = 1); ST3B (*n* = 2)
	Female	1/19 (5.3)	ST3A (*n* = 1)
	Subtotal	4/38 (10.5)	ST3A (*n* = 2); ST3B (*n* = 2)
Pishan	Male	5/16 (31.3)	ST1C (*n* = 1); ST2C (*n* = 2); ST3A (*n* = 2)
	Female	2/21 (9.5)	ST2C (*n* = 1); ST3A (*n* = 1)
	Subtotal	7/37 (18.9)	ST1C (*n* = 1); ST2C (*n* = 3); ST3A (*n* = 3)
Lop	Male	5/43 (11.6)	ST1A (*n* = 1); ST1B (*n* = 2); ST3A (*n* = 1); ST3B (*n* = 1)
	Female	9/66 (13.6)	ST1A (*n* = 1); ST1B (*n* = 5); ST1F (*n* = 1); ST2D (*n* = 1); ST3A (*n* = 1)
	Subtotal	14/109 (12.8)	ST1A (*n* = 2); ST1B (*n* = 7); ST1F (*n* = 1); ST2D (*n* = 1); ST3A (*n* = 2); ST3B (*n* = 1)
Total	Male	44/299 (14.7)	ST1A (*n* = 10); ST1B (*n* = 6); ST1C (*n* = 4); ST2A (*n* = 1); ST2C (*n* = 2); ST3A (*n* = 13); ST3B (*n* = 8)
	Female	43/310 (13.9)	ST1A (*n* = 8); ST1B (*n* = 7); ST1D (*n* = 1); ST1E (*n* = 1); ST1F (*n* = 1); ST2A (*n* = 2); ST2B (*n* = 1); ST2C (*n* = 1); ST2D (*n* = 1); ST3A (*n* = 16); ST3B (*n* = 4)
		87/609 (14.3)	ST1A (*n* = 18); ST1B (*n* = 13); ST1C (*n* = 4); ST1D (*n* = 1); ST1E (*n* = 1); ST1F (*n* = 1); ST2A (*n* = 3); ST2B (*n* = 1); ST2C (*n* = 3); ST2D (*n* = 1); ST3A (*n* = 29); ST3B (*n* = 12)

*Note: P *> 0.05 for the difference in prevalence of *Blastocystis* between males and females

### Distribution of *Blastocystis* subtypes

The DNA sequence analyses of the *SSU* rRNA gene products revealed the presence of three *Blastocystis* subtypes, ST1 (38/87), ST2 (8/87) and ST3 (41/87) (Table 1). Subtype ST3 was dominant in most of the counties surveyed, with ST1 appearing to be the predominant subtype in the Lop county and only ST1 being identified in the Poskam county (Table 1). The predominance of subtype ST3 was found both in males and females, followed by subtypes ST1 and ST2 (Table 1).

### The polymorphic nature of the *SSU* rRNA gene of *Blastocystis*

**Fig. 2:**
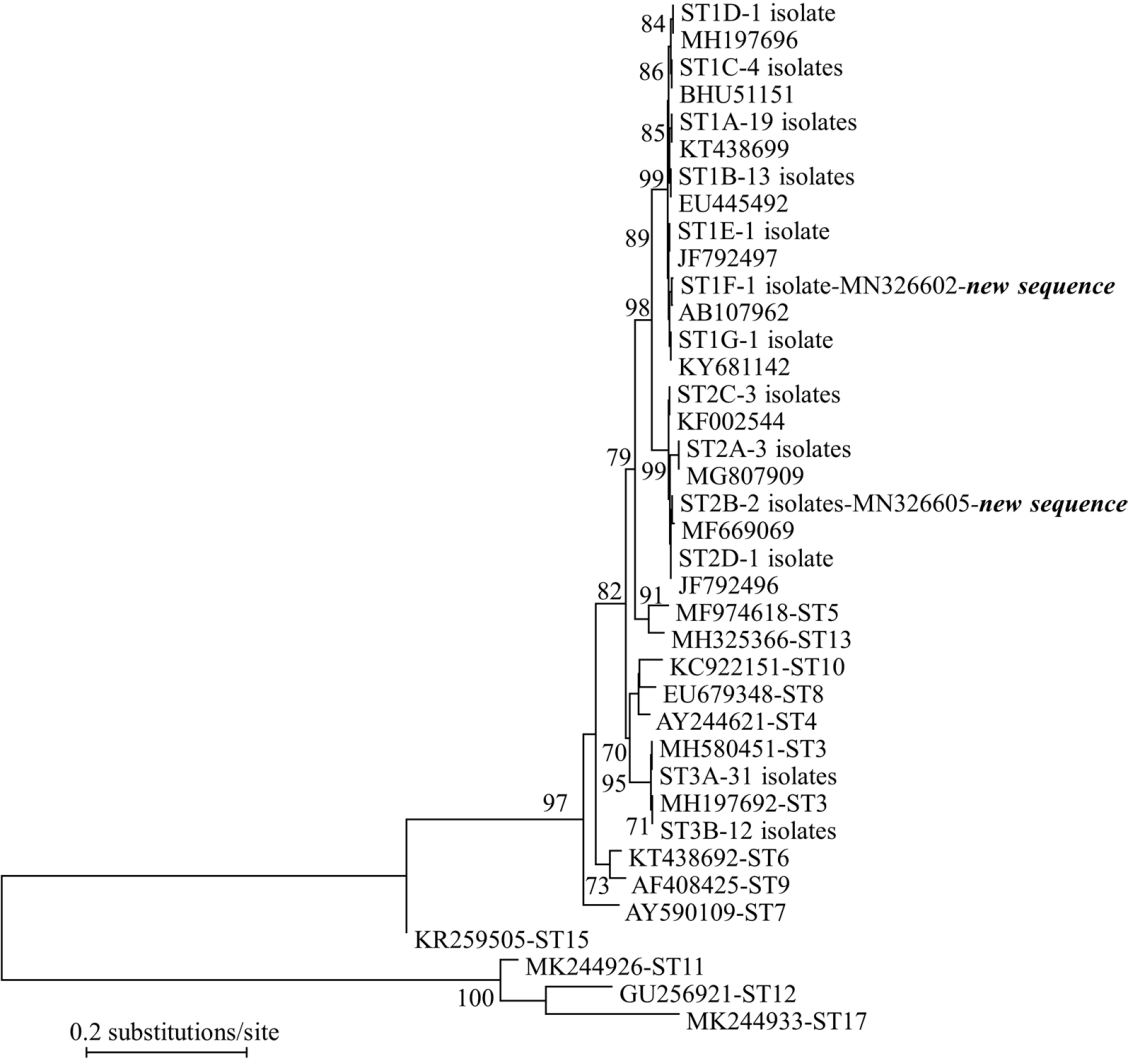
Phylogenetic relationships among representative sequences of the *Blastocystis* small subunit ribosomal RNA (*SSU* rRNA) genes obtained from China, using the neighbor-joining method. Bootstrap values greater than 70% from 1000 pseudo replicates are shown

Seven variations of the ST1 subtype were identified by sequence alignment analysis, with the similarity ranging between 99.0–99.7%. ST1A, comprising 19 out of 40 ST1 subtypes, was the dominant sequence type, followed by the variations ST1B (*n* = 13) and ST1C (*n* = 4). The variations ST1D, ST1E, ST1F and ST1G were detected in only one sample each (Table 1). Likewise, nine ST2 isolates produced four variations (ST2A to ST2D), with a similarity of 99.5–99.8%. In contrast, little difference was observed in the ST3 subtype. Forty-three ST3 isolates formed two variations (ST3A and ST3B) and only one nucleotide difference was noticed between them. Among these types, ST1F and ST2B represented new variations. Phylogenetic analysis also confirmed the polymorphic nature of the *SSU* rRNA gene of *Blastocystis* isolates in this study (Fig. 2).

## Discussion

*Blastocystis* infection was shown to be prevalent in kindergarten children in the study area; the overall infection rate of 14.3% was identical to the 14.3% (1/7) prevalence reported in children < 5 years of age in Yunnan Province [23]. Nevertheless, the rate is significantly lower than that in some developing countries, such as Nigeria (83.9%, 167/199; 2–14 years of age) and Turkey (38.0%, 115/303; 3–13 years of age) [24, 25]. In China, the average infection rate of *Blastocystis* in humans was 3.4% (3625/107,695) [20], with the highest prevalence being 43.3% (215/497) in Guangxi Province [26]; the lowest rate was 0.80% (85/10,652) in Fujian Province [27]. Previous studies have suggested that the variation in the prevalence of human *Blastocystis* infection may be closely related to the immune status and age of the hosts and different geographical locations [23, 27–30]. In addition, infection with *Blastocystis* was associated with drinking unboiled water in a hill village in Yunnan Province, China [23]. An outbreak of human *Blastocystis* infection was observed following ingestion of contaminated running water, and over 1122 patients with diarrhea were identified [31]. There was no significant difference in *Blastocystis* prevalence associated with sex in this study, which was similar to the findings of previous studies [23, 28].

In this study, subtypes ST1 (*n* = 38), ST2 (*n* = 8) and ST3 (*n* = 41) were identified by using sequence analysis of the *SSU* rRNA gene, with the predominance of subtype ST3 being found both in males and females, followed by subtypes ST1 and ST2. This distribution of *Blastocystis* subtypes is similar to that found in most countries of the world [14, 15]. In contrast, the most dominant subtype in Spain and Denmark was reported to be ST4 [18, 32]. It is reported that the prevalence of *Blastocystis* is affected by various epidemiological and demographic factors such as climate, geography, cultural habits, and exposure to reservoir hosts [15].

In China, six *Blastocystis* subtypes (ST1 to ST6) have been identified and most of the samples represented single-subtype infections, while mixed infections were also observed (such as ST1 + ST2, ST1 + ST3, ST2 + ST3, ST3 + ST5), and some subtypes were novel [23, 29, 30, 33]. Among them, ST3 (62%, 186/300) is the dominant subtype and has been identified in almost all reports from China [23, 29, 30, 33]. In addition, ST3 is also a commonly identified subtype in humans in Europe, Africa, Oceania and the Middle East [14, 34–36].

As observed in the present study, genetic diversity varies dramatically among *Blastocystis* subtypes, as well as at the intra-subtype level. However, whether genetic diversity is associated with symptoms or not is far from clear. Dogruman et al. [37] reported that ST2 was associated with asymptomatic infection. In another study by the same authors, ST3 was found to be the most common subtype in both symptomatic and asymptomatic groups, but there was no association between *Blastocystis* subtypes and gastrointestinal symptoms [38]. Similarly, three studies conducted in Thailand and Turkey suggested that ST3 was the most frequent subtype but did not observe a significant relationship between this subtype and gastrointestinal symptoms [25, 39, 40].

## Conclusions

A *Blastocystis* infection rate of 14.3% was identified in kindergarten children in southern Xinjiang, China, with no significant difference in *Blastocystis* prevalence between males and females. Three *Blastocystis* subtypes (ST1 to ST3) were identified by PCR and ST3 was the predominant subtype; in addition, genetic polymorphism was observed within subtypes. More extensive studies in both humans and animals in different areas are needed to better characterize the transmission of *Blastocystis*.

## Data Availability

Representative nucleotide sequences generated in this study were deposited in the GenBank database under the Accession Numbers MN326597–MN326609.

## Abbreviations

PCR: polymerase chain reaction
*SSU* rRNA: small subunit ribosomal RNA
ST: sequence type
